# Supplementary material for: The interactome of intact mitochondria by cross-linking mass spectrometry provides evidence for coexisting respiratory supercomplexes
Source: Mol Cell Proteomics. 2017 Dec 8;17(2):216–32. doi: 10.1074/mcp.RA117.000470 (PMC5795388; doi:10.1074/mcp.RA117.000470)
Supplement: Supplemental Data [file supp_RA117.000470_133922_0_supp_23978_fzffwf.zip › spectra_annotation/mito_spectra_annotation/3-1-9-1-2-1.pdf]

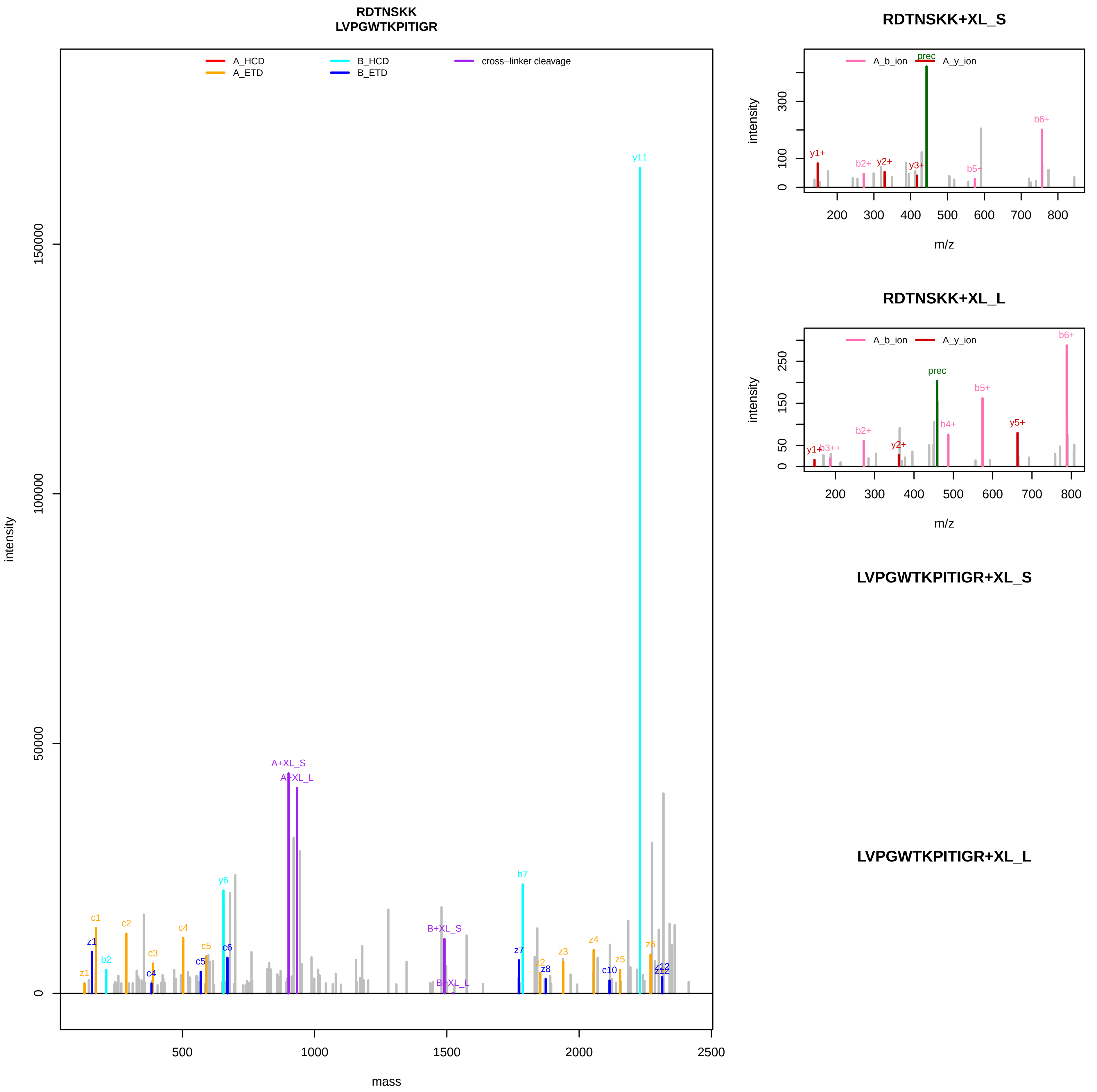
RDTNSKK
LVPGWTKPITIGR
A_HCD
A_ETD
B_HCD
B_ETD
cross−linker cleavage
intensity
mass
y11
A+XL_S
A+XL_L
B+XL_S
B+XL_L
y6
b7
b2
c1
c2
c3
c4
c5
c6
z1
z2
z3
z4
z5
z6
z7
z8
z12
c10
RDTNSKK+XL_S
A_b_ion
A_y_ion
prec
y1+
b2+
y2+
y3+
b5+
b6+
m/z
RDTNSKK+XL_L
b3++
b4+
y5+
LVPGWTKPITIGR+XL_S
LVPGWTKPITIGR+XL_L
